# Induction of alveolar and bronchiolar phenotypes in human lung organoids

**DOI:** 10.14814/phy2.14857

**Published:** 2021-06-10

**Authors:** Laurence Hoareau, Agnete S. T. Engelsen, Marianne Aanerud, Maria Paula Ramnefjell, Pirjo‐Riitta Salminen, Fabian Gärtner, Thomas Halvorsen, Helge Ræder, Mariann H. L. Bentsen

**Affiliations:** ^1^ Department of Clinical Science Faculty of Medicine University of Bergen Bergen Norway; ^2^ Department of Pediatrics Haukeland University Hospital Bergen Norway; ^3^ Department of Biomedicine University of Bergen Bergen Norway; ^4^ Centre for Cancer Biomarkers University of Bergen Bergen Norway; ^5^ Department of Thoracic Medicine Haukeland University Hospital Bergen Norway; ^6^ Department of Pathology Haukeland University Hospital Bergen Norway; ^7^ Heart Department Section of Cardiothoracic Surgery Haukeland University Hospital Bergen Norway

**Keywords:** 3D model, lung epithelium, organoids

## Abstract

Patient‐derived organoids have revolutionized biomedical research and therapies by "transferring the patient into the Petri dish". In vitro access to human lung organoids representing distal lung tissue, i.e. alveolar organoids, would facilitate research pertaining to a wide range of medical conditions and might open for a future approach to individualized treatment.We propose a protocol to derive a single human lung biopsy towards both alveolar and bronchiolar organoids. By modulating Wnt pathway, we obtained a differential gene expression of the main markers for both subtypes, such as a higher expression of surfactant protein C in alveolar organoids or a higher expression of mucine 5AC in bronchiolar organoids. Although the specific cell enrichment was not complete, the differentiation was observed as early as passage 1 based on morphology, and confirmed by QPCR and histology at passage 2. These results are consistent with a functional specification of lung epithelium towards both alveoli‐ and bronchi‐enriched organoids from first passages

## Graphical abstract

We propose a protocol to derive a single human lung biopsy towards alveolar and bronchiolar organoids. By modulating Wnt pathway, we obtained a differential gene expression of the main markers for both subtypes, suggesting a functional specification.

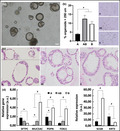

## 1 INTRODUCTION

Patient‐derived organoids have revolutionized biomedical research and therapies by “transferring the patient into the Petri dish”. Current protocols to establish organoids from dissociated human airway epithelium support mainly the differentiation towards bronchiolar organoids with the four cell lineages: ciliated, goblet, club and basal cells (Sachs et al., 2019; Zhou et al., 2018). Alveolar organoids, containing alveolar type 1 and type 2 cells, were first established from iPSCs, lung cancer cells or with cell line co‐cultures (Barkauskas et al., 2013; Lee et al., 2017). Protocols for the establishment of alveolar organoids derived from human material are also available, but they require cell enrichment and co‐culture with feeder cells (Barkauskas et al., 2013; Katsura et al., 2020; Youk et al., 2020; Zacharias et al., 2018). More direct protocols for *in vitro* access to human lung organoids representing distal lung tissue would facilitate research pertaining to a wide range of medical conditions and might open for a future approach to individualized treatment.

Our main goal was to derive three differentiated organoid subtypes from a single biopsy of human lung tissue: alveolar, bronchiolar and broncho‐alveolar organoids, based on feeder‐free chemically defined culture conditions and without any cell selection.

## 2 METHODS

This study was approved by the Regional Ethics Committee (REK Vest of Norway, approval #66610). A specimen of healthy lung was obtained from a male patient undergoing lobectomy. The sample (0.47 cm^3^) was maintained on ice and processed within one hour after sampling. Tissue was washed twice in PBS and minced by scalpels. Tissue pieces were collected in aDMEM/F12 and incubated with 2 mg/ml collagenase NB4 (Serva) for 1h30, at 37°C under rotation.

Tissue fragments were then pipetted vigorously, filtered through a 70 µm mesh and centrifuged 5 min at 400 *g*. The pellet was resuspended in growth factor reduced Matrigel (Corning) and plated in culture vessels. After gelling, domes were covered with aDMEM/F12 complemented with B27 (Gibco), N‐acetyl‐cysteine 1,25 mM (Sigma‐Aldrich), Noggin 100 ng/ml, R‐Spondin‐1 500 ng/ml, FGF7 25 ng/ml, FGF10 100 ng/ml (all from Peprotech), A83‐01 500 nM, SB202190 500 nM (both from BioGems). Y27632 5 µM (BioGems) was added the first day of culture and after each passage. To induce the different phenotypes, nicotinamide 5 mM (Sigma‐Aldrich) and CHIR99021 3 µM (BioGems) were added for the alveolar culture, nicotinamide 5 mM for the bronchiolar culture and CHIR99021 3 µM for the broncho‐alveolar culture. In order to increase the viability, organoids were maintained in the well‐characterized bronchiolar differentiation medium (Sachs et al., 2019) for one week before starting the three different cultures.

Organoids were incubated at 37°C, 5% CO_2_. Medium was changed twice a week and organoids were passaged every two weeks (d14 and d28).

At day 45 (after 2 passages), organoids were rinsed three times in PBS^−/−^ prior to further processing. For gene expression analysis, organoids were dissociated in lysis buffer and processed with NORGENE RNA extraction kit, according to the manufacturer's instructions. RNA was quantified, and 1 µg was reverse transcribed with the SuperScriptII reverse transcriptase, according to the manufacturer's instructions.

Real Time Quantitative PCR (Q‐PCR) analysis was performed using 30 ng of cDNA per reaction, along with the TaqMan Master Mix from Applied BioSystem. Samples were run in triplicates and results were normalized to GAPDH expression. Quantification was done using the ΔΔC_T_ method. Primers and probes were selected from the TaqMan Gene Expression Assays; housekeeping gene GAPDH: Hs02758991_g1, surfactant protein C: Hs00161628_m1, podoplanin: Hs00366766_m1, mucine 5AC: Hs01365616_m1, Forkhead box J1: Hs00230964_m1, keratin 5: Hs00361185_m1, secretoglobulin 3A2: Hs00369678_m1.

For histological analysis, organoids were fixed in formalin 3.7% for 24 hours, dehydrated and embedded in paraffin wax according to standard procedures. After sectioning (6 µm), paraffin wax was removed by 3 × 5 min washes in xylene, and rehydrated in alcohol (3 × 100%, 1 × 96%, 1 × 70% and tap water) prior to staining with hematoxylin and eosin according to standard histopathological procedures.

## 3 RESULTS AND DISCUSSION

**FIGURE 1:**
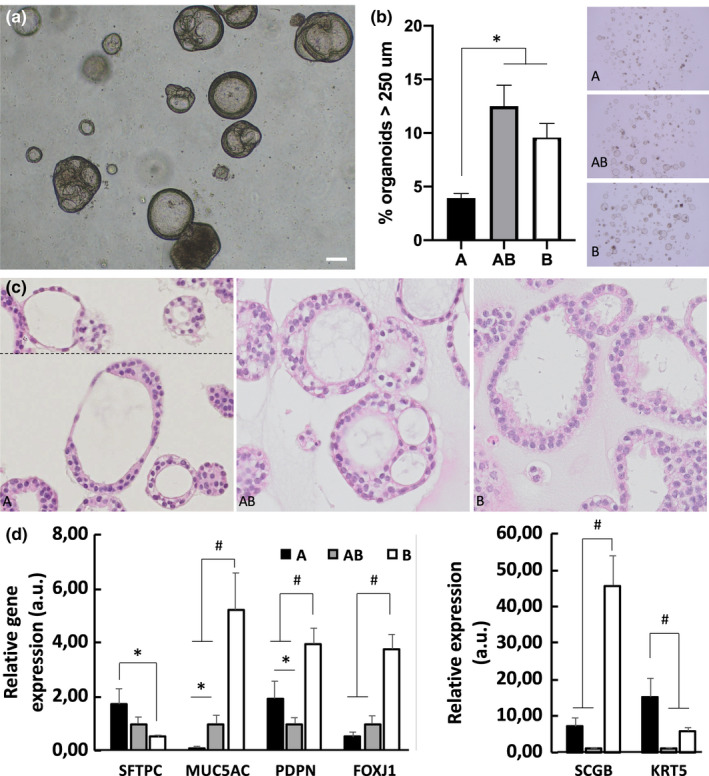
(a) Organoids from bronchiolar differentiation at d14, scale bar: 100 µm. (b) Organoid size at d28, average from 2 domes per condition (*p < 0.05) and representative picture of one dome per condition; A: alveolar differentiation, AB: broncho‐alveolar differentiation, B: bronchiolar differentiation. (c) H&E staining at d45, square size: 200 µm. (d) Relative gene expression analysis at d45, normalized to AB condition (*p < 0.05, ^#^ *p* < 0.01). Left: surfactant protein C (SFTPC), mucine 5AC (MUC5AC), podoplanin (PDPN), transcription factor FOXO1 J (FOX1 J)

After three days of culture, multicellular structures sized 30 to 80 µm in diameter and with a prominent lumen were detected (data not shown). Irrespective of the differentiation medium used, we observed heterogeneity within the organoid cultures with respect to different sizes and morphologies, such as single spheroids as well as more complex structures like multilocular units (Figure 1a).

Organoids grown in the alveolar culture medium were consistently smaller than the ones grown in the bronchiolar and broncho‐alveolar culture media. At d28, only 4% of the alveolar‐enriched organoids were larger than 250 µm, compared to more than 10% for the two other phenotypes (Figure 1b). The histological assessment confirms the smaller size of alveolar organoids. While most of them are characterized by proliferative tissue of immature appearance, organoids composed of a single layer of more differentiated cells are also apparent. Some of these cells are characterized by their flat squamous appearance with characteristic elongated nuclei suggesting alveolar type 1 cells (Figure 1c). The two other phenotypes more frequently consist of several cell layers and multilocular structures.

At the gene expression level, we found that surfactant protein C, marker of alveolar type 2 cells, was expressed 3,5‐fold higher in alveolar organoids, compared to bronchiolar organoids (Figure 1d), suggesting functional specification of alveolar organoids. Likewise, markers for goblet cells (mucine 5AC), club cells (secretoglobulin 3A2) and ciliated cells (transcription factor FOXO1J) were expressed 6‐ to 50‐fold higher in bronchiolar organoids, suggesting functional specification of bronchiolar organoids. The specification is also observed in histologic sections, as cytoplasm from organoids grown in bronchiolar and broncho‐alveolar media are more eosinophilic, suggesting mucus production (Figure 1c). In addition, we also observed pulsatile cilia in some organoids in culture, demonstrating a central feature of functionality of the ciliated cells (data not shown).

These results are consistent with a functional specification of lung epithelium towards both alveoli‐ and bronchi‐enriched organoids from passage 2. However, the higher expression of keratin 5 in alveolar organoids would also suggest the presence of basal cells and that these organoids are not yet terminally differentiated. Podoplanin has been shown to be expressed mainly in both alveolar type 1 cells and basal cells. Our results demonstrate a higher expression of podoplanin in bronchiolar organoids, which doesn't match with the expression profile for keratin 5, another marker of basal cells. This may suggest that the current protocol needs further optimization, and it may also reflect the fact that the organoids are still in development and not terminally differentiated as e.g. podoplanin has been shown to be expressed by numerous cells during lung morphogenesis (Ugorski et al., 2016).

We started our protocol with a well‐defined bronchiolar induction medium (Sachs et al., 2019). In the same way that three different types of organoids were established from stomach epithelium (pit‐ and gland‐domains were stimulated with Wnt molecules, gland domains with Wnt molecules and nicotinamide, and pit domains with nicotinamide (Bartfeld et al., 2015)), we used a potent Wnt activator: CHIR99021, a GSK‐3 inhibitor, and nicotinamide to induce alveolar phenotype and confirmed the results obtained recently with Wnt3a (Salahudeen et al., 2020).

## 4 CONCLUSION

We succeeded in generating three different airway organoid subtypes from a distal lung biopsy: alveolar, broncho‐alveolar and bronchiolar organoids. Although the specific cell enrichment was not complete, the differentiation was observed as early as passage 1 based on morphology, and confirmed by QPCR and histology at passage 2. In the context of personalized medicine, expanding our knowledge on early passaged‐organoids would benefit patients. When survival is a matter of time, the sooner organoids may be used for drug assessment in cancer therapy or for grafting or disease‐modelling in regenerative medicine, the faster health measures can be implemented for patients.

## CONFLICT OF INTEREST

Authors have no conflict of interest.

## AUTHORS’ CONTRIBUTIONS

All authors conceived and designed the research. LH, PRS, and MPR performed experiments. LH, ASTE, and MPR analyzed the data. LH, MHLB, ASTE, HR, and MPR interpreted results of experiments. MPR, ASTE, and LH prepared the figures. LH, ASTE, MHLB, HR, and TH drafted the manuscript. All authors edited and revised the manuscript and approved the final version.
